# Meningitis in HIV-positive patients in sub-Saharan Africa: a review

**DOI:** 10.7448/IAS.17.1.19184

**Published:** 2014-10-10

**Authors:** Jennifer A Veltman, Claire C Bristow, Jeffrey D Klausner

**Affiliations:** 1 Division of Infectious Diseases, University of California Los Angeles, Los Angeles, CA, USA; 2 Department of Epidemiology, Fielding School of Public Health, University of California Los Angeles, Los Angeles, CA, USA

**Keywords:** meningitis, HIV/AIDS, adult, sub-Saharan Africa

## Abstract

**Introduction:**

Meningitis is one of the leading causes of death among patients living with HIV in sub-Saharan Africa. There is no widespread tracking of the incidence rates of causative agents among patients living with HIV, yet the aetiologies of meningitis are different than those of the general population.

**Methods:**

We reviewed the scientific literature published in PubMed to determine the incidence rates of meningitis among hospitalized people living with HIV in sub-Saharan Africa and report our findings from seven studies across sub-Saharan Africa.

**Results:**

We found high rates of cryptococcal meningitis (19–68%). Tuberculous meningitis was lower (1–36%), although some centres included possible cases as “other” meningitis; therefore, this may not be a true representation of the total cases. Pyogenic meningitis ranged from 6 to 30% and “other” meningitis ranged from 7 to 28% of all reported cases of meningitis. Mortality rates ranged from 25 to 68%. This review describes the most common aetiologies and provides practical diagnostic, treatment and prevention considerations as they apply to the individual living with HIV in sub-Saharan Africa.

**Conclusions:**

Diagnosis is often limited, and wider availability of accurate and low-cost laboratory diagnostics is desperately needed for prompt diagnosis and initiation of appropriate treatment. Wider acceptance and adoption of available preventative modalities can decrease the incidence of potentially fatal central nervous system infections in African patients living with HIV.

## Introduction

Infectious meningitis is an extremely serious condition and is one of the leading causes of morbidity and mortality in HIV-negative and HIV-positive persons living in sub-Saharan Africa [1,2]. There are a variety of organisms that cause meningitis, such as meningococcus; an increasing prevalence of *Mycobacterium tuberculosis* and *Cryptococcus neoformans* has been reported in association with the HIV epidemic, yet there is no widespread reporting of the meningitis aetiologies among people living with HIV in sub-Saharan Africa [3,4]. Whatever data that exist on the aetiology of meningitis among this group come from small, hospital-based cohort studies. Prompt diagnosis is critical for initiation of proper treatment but is often challenging, especially in a setting with limited resources. This review describes the most common aetiologies and provides practical diagnostic, treatment and prevention considerations as they apply to the individual living with HIV in sub-Saharan Africa.

## Methods

One investigator reviewed the published scientific literature by using PubMed to determine the incidence rates of meningitis among adults living with HIV in sub-Saharan Africa. We searched the published medical literature from November 1987 until March 2014 for eligible articles, using the search terms “HIV,” “meningitis,” “adult,” and “Africa.” Our primary outcome of interest was the prevalence rate of meningitis among patients living with HIV in sub-Saharan Africa. Inclusion criteria were: (1) involving adult patients; (2) involving health centres located in sub-Saharan Africa; (3) reported prevalence of a specific aetiology of meningitis among persons living with HIV, or reported results from which prevalence rates among persons living with HIV could be calculated; and (4) utilized a prospective or retrospective cohort study design. Exclusion criteria included: non-English studies.

Regarding statistical analysis, prevalence rates were tabulated and means were calculated using STATA statistical software.

## Results

Our initial literature search yielded 242 citations. From those, we identified seven reports that met our eligibility criteria. The criteria for diagnosis most frequently used were the clinical signs of meningeal irritation, such as photophobia, neck rigidity, vomiting, headaches, fever, altered mental status and new-onset seizures (symptoms reported in four of seven studies). The diagnostic criteria for pyogenic meningitis always involved bacterial culture. In six of seven studies, a positive Gram stain was also included. In two of seven studies, a bacterial antibody test was used; and in six of seven studies, cerebrospinal fluid (CSF) findings of elevated white blood cell count with >50% neutrophils were also included. The criteria for tuberculous meningitis always included mycobacterial culture: acid-fast bacilli (AFB) smear was included in three of seven studies, and mycobacterial polymerase chain reaction was included in one of six studies. One study used consistent clinical picture and response to anti-TB medications as diagnostic criteria. In three of seven studies, patients with TB isolated in another body location with signs and symptoms of meningitis were included. For the diagnostic criteria for cryptococcal meningitis, all studies used fungal culture in their diagnostic criteria. All studies performed a cryptococcal antigen (CrAg) on the specimens, but only five of seven studies included these findings in the diagnostic criteria. Five studies performed India ink on CSF samples, but only four of these studies used the findings as part of their diagnostic criteria. Patients who met criteria for meningitis but did not meet diagnostic criteria for pyogenic, tuberculous or cryptococcal meningitis were classified as “other.”

Table 1 shows the rates of meningitis infection among patients living with HIV, ages >18 years, from seven studies. High rates of cryptococcal meningitis were seen in all studies, ranging from 19 to 68% of all reported cases of meningitis. The prevalence of tuberculous meningitis was lower (1–36%), although some centres included possible cases as “other” meningitis; therefore, this may not be a true representation of the total cases. Pyogenic meningitis ranged from 6 to 30% and other meningitis ranged from 7 to 28% of all reported cases of meningitis.

**Table 1:** Aetiology of meningitis among patients living with HIV in seven African medical centres

Country	Author, year	Total cases of meningitis	Cryptococcal meningitis, *n* (%)	Pyogenic meningitis, *n* (%)	Tuberculous meningitis, *n* (%)	Other meningitis^a^, *n* (%)
Central African Republic	Bekondi, 2006	215	84 (39.1)	66 (30.7)	3 (1.4)	62 (28.8)
Zimbabwe	Hakim, 2000	170	80 (47.1)	25 (14.7)	21 (12.4)	44 (25.9)
South Africa	Bergemann, 1996	106	37 (34.9)	10 (9.4)	39 (36.8)	20 (18.9)
South Africa	Jarvis, 2010	492	337 (68.5)	29 (5.9)	126 (25.6)	n/a^b^
Malawi	Cohen, 2010	235	110 (46.8)	48 (20.4)	41 (17.4)	36 (15.3)
South Africa	Schutte, 1999	44	22 (50.0)	3 (6.8)	16 (36.4)	3 (6.8)
South Africa	Silber 1999	41	8 (19.5)	4 (9.8)	9 (21.9)	20 (48.8)
Total		1303	678 (52.0)	185 (14.2)	255 (19.6)	185 (14.2)

a Other = lymphocytic, mononuclear, neuro-syphilitic, post-surgical and viral aetiologies;

b unable to calculate, as the original article did not include HIV status percentiles for other meningitis aetiologies.

Overall, meningitis patients living with HIV had 2–10 times higher mortality rates than meningitis patients who were HIV-negative. In Central African Republic, in-hospital mortality was 16.7% for HIV-negative patients and 57% for patients living with HIV, with the highest mortality seen in those patients with cryptococcal meningitis and pyogenic meningitis [5]. In Zimbabwe, in-hospital mortality in patients living with HIV and meningitis was highest for pyogenic meningitis (68%) and overall was 45.8%. The mortality rate was not given for HIV-negative patients [6]. In Pretoria, South Africa, 25% of the meningitis patients living with HIV died at the end of the four-year study period, a rate that was more than double that of the HIV-negative/not tested group (11%) [4]. In Soweto, South Africa, there was no difference in mortality rates between patients living with HIV and those who are HIV-negative: overall mortality was 31% [3]. The Gold Fields, South Africa, study reported a six-month mortality rate of 47.4% among HIV-positive patients and 5.3% in HIV-negative patients [7]. The Cape Town, South Africa, study and the Malawian study did not report mortality results [8,9].

## Discussion

This review provides an updated, systemic review of the prevalence of specific aetiologies of meningitis among persons with HIV infection in sub-Saharan Africa. We found that the most common form of meningitis among patients living with HIV in sub-Saharan Africa was cryptococcal meningitis. No large, prospective, population-based studies exist on the aetiologic incidence of meningitis among the general population in sub-Saharan Africa; however, the data that exist indicate that the most common aetiologies among the general population depend on the HIV prevalence in that region. In areas with low HIV prevalence, bacterial causes such as meningococcal meningitis are most prevalent; however, in areas of high HIV prevalence, cryptococcal and tuberculous meningitis have been found to be most prevalent, which correlates with our findings when aetiologies among only HIV-positive patients are examined [6,8,10]. These findings have broad implications for health care centres and increase the diagnostic and management complexity, as cryptococcal meningitis and tuberculous meningitis can be very difficult to diagnose and treat. Overall, mortality rates among HIV-positive patients were also higher compared to those of HIV-negative patients, indicating a larger role for prevention of these diseases.

Treatment of meningitis and its complications has several components. Those include: identifying the organism in a timely fashion; finding and instituting appropriate antibiotic therapy; managing increased intracranial pressure; and managing neurological complications (e.g. stroke and seizures) and systemic complications (e.g. syndrome of inappropriate antidiuretic hormone secretion (SIADH) and sepsis). Delaying or mismanaging any of those components can result in significant mortality and morbidity, including hearing loss, focal neurological deficits, learning disorders and seizures. We have set out to summarize the most common causes of meningitis in individuals living with HIV in sub-Saharan Africa and present important diagnostic, treatment and prevention considerations.

### Bacterial meningitis

#### Streptococcus pneumoniae

Pneumococcal meningitis, caused by the bacterium *S. pneumoniae*, is a serious and frequently life-threatening disease. It has been established in several studies, both internationally and in the United States, that patients living with HIV have a much higher risk of developing pneumococcal meningitis than the general population, with relative risks reported between 20 and 150 times higher [11,12]. The exact mechanism for that increased risk is not known.

In the African setting, the mortality rate associated with pneumococcal meningitis is especially high compared to rates in high-income countries [13]. In Malawi, the mortality rate among patients with pneumococcal infection was 53.4%, of whom 87% were living with HIV [14]. Similar rates were seen in Senegal with a pneumococcal meningitis mortality rate of 69.8%, of whom only 12% were living with HIV [15]. This is compared to a Spanish cohort of patients living with HIV who had a mortality rate of 11% [16]. Possible reasons for this striking difference include delays in presentation and clinical management, limited access to antiretroviral treatment among the African population as well as suboptimal resuscitation and intensive care support [14].

Pneumococcal meningitis most often presents with the classic triad of fever, neck stiffness and altered mental status [16]. Patients with pneumococcal meningitis can also present with sensitivity to light, headaches and vomiting [17]. Atypical presentations have been observed, especially in patients living with HIV, and they include seizures as the presenting symptom [5,11]. Patients with pneumococcal meningitis were more likely to have distant foci of infection, including pneumonia, sinusitis and otitis (63%), compared to other aetiologies of meningitis in one large study [16].

The first-line treatment in sub-Saharan Africa is intravenous or intramuscular ceftriaxone, cefotaxime or chloramphenicol and should be initiated as soon as a presumptive diagnosis of bacterial meningitis is made [18]. In a recent meta-analysis of antibiotic susceptibility among *S. pneumoniae* in Africa from 1978 to 2011, 23.8% of isolates were found to be non-susceptible to penicillin and 5.7% were non-susceptible to chloramphenicol. Ceftriaxone or cefotaxime resistance was only seen in 3.1% of pneumococcal isolates, supporting the continued use of third-generation cephalosporins as a first-line antibiotic treatment [19]. Not surprisingly, considering the widespread use of trimethoprim-sulphamethoxazole in sub-Saharan Africa, resistance was seen in 51.6% of isolates.

Even when treated with effective antibiotics, pneumococcal meningitis continues to have high rates of mortality and neurological morbidity including hearing loss, hemiparesis, epilepsy, ataxia and cognitive disability. Adjunctive steroid treatment is often given to attenuate inflammation in the subarachnoid space in an attempt to decrease the morbidity and mortality of this disease. In a recent meta-analysis, dexamethasone as adjunctive treatment in high-income countries did show reduction in severe hearing loss (RR, 0.51; 95% CI, 0.35 to 0.73), any hearing loss (RR, 0.58; 95% CI, 0.45 to 0.73) and short-term neurological sequelae (RR, 0.64; 95% CI, 0.48 to 0.85), as well as a trend towards mortality benefit (RR, 0.81; 95% CI, 0.65 to 1.05; *p =* 0.10). Benefits in low-income countries were not seen in mortality (RR, 1.00; 95% CI, 0.88 to 1.14), severe hearing loss (RR, 0.99; 95% CI, 0.72 to 1.38), any hearing loss (RR, 0.91; 95% CI, 0.78 to 1.06) or short-term neurological sequelae (RR, 1.03; 95% CI, 0.81 to 1.31). Patients studied in low-income countries were often malnourished and admitted in a later state of disease, which may lessen the protective effect of steroids. Patients in those studies also had higher HIV infection rates and often received suboptimal antibiotics, which may also have led to the lack of benefit seen in this population. In a Vietnamese study with a <1% HIV prevalence rate, mortality benefit was seen with steroids, but because of the low HIV prevalence rate, these findings cannot be applied to African patients living with HIV [20]. Because of the lack of benefit seen in African studies, steroids are not generally recommended in sub-Saharan Africa [21–23]. Oral glycerol as an adjunctive treatment was recently studied in an African context and shown to have no benefit, with a trend towards increased mortality [24].

High rates of pneumococcal meningitis in Africa with associated high mortality rates make this a prime target for prevention by immunization. However, the impact of polysaccharide pneumococcal vaccine (PPV) on people living with HIV who are not on antiretroviral therapy (ART) remains controversial. Retrospective cohort studies suggest benefit in patients living with HIV in the United States; however, a randomized controlled trial showed no benefit in the African context, and therefore the PPV is not recommended alone [25–27]. PPV is recommended in combination with the pneumococcal conjugate vaccine (PCV) to expand the coverage of potential disease-causing serotypes [28]. That recommendation is extrapolated from a randomized controlled trial using seven-valent pneumococcal conjugate vaccine (PCV-7) in Malawian adults (88% living with HIV) who had recovered from an episode of invasive pneumococcal disease; that vaccine prevented 74% of recurrent episodes of invasive pneumococcal disease [29]. There are no current World Health Organization (WHO) recommendations for the use of pneumococcal vaccines in the care of adults living with HIV; however, WHO recommends the inclusion of PCVs in childhood immunization programmes worldwide, which has the potential of offering secondary benefit to adults living with HIV through herd immunity. The uptake of that recommendation has been limited, with only 21% of African children <5 years of age in 2012 receiving three doses of PCV [28,30]. PCVs are an effective and safe preventive strategy, and increasing their availability, both in the general population and potentially as part of routine HIV care, should be a top priority.

#### Neisseria meningitidis

Meningococcal meningitis is generally thought to cause epidemics in the traditionally defined “meningitis belt” (from Senegal in the west to Ethiopia and Sudan in the east). There is evidence, however, that this meningitis belt may be expanding as a result of climate change to include portions of Southern and Eastern Africa [31]. Outbreaks most commonly occur during the dry season, during which time it is hypothesized that aerosolized dust damages lung mucosa resulting in increased coughing and transmissibility [32].

Several reports from Africa have concluded that HIV infection does not increase susceptibility to meningococcal disease [33–36]. That lack of association has not been found in all studies, however, and a recent study from South Africa showed that the incidence of meningococcal disease in HIV-positive individuals was elevated in all age groups compared to HIV-negative individuals (relative risk, 11.3; 95% CI, 8.9–14.3). The study also showed excess mortality among individuals living with HIV (20% vs. 11%; odds ratio (OR), 2.1; 95% CI, 1.1–3.9) [37].

Meningococcal meningitis has a similar presentation to pneumococcal meningitis, with neck stiffness, fever, sensitivity to light, altered mental status, headaches and vomiting. The presentation can be much more severe in bacteraemic patients who may present with characteristic skin manifestations, such as petechiae and palpable purpura, loss of limbs due to tissue necrosis, adrenal infarction leading to adrenal insufficiency (Waterhouse-Friderichsen syndrome), purpura fulminans, and disseminated intravascular coagulation leading to rapid circulatory collapse.

Treatment consists of hospital care and prompt intravenous antibiotic therapy. Antibiotics recommended by WHO include intravenous penicillin, ampicillin, chloramphenicol or ceftriaxone. In cases of epidemics where resources are limited, a single dose of intravenous ceftriaxone or intramuscular, long-acting (oily) chloramphenicol has been shown to be effective [38]. The most recent review of *N. meningitidis* isolates from sub-Saharan Africa was published in 2009 and showed that all *N. meningitides* isolates were susceptible to ceftriaxone, chloramphenicol and ciprofloxacin. No isolate produced β-lactamase. Only three isolates (2%) displayed reduced susceptibility to penicillin G, thus supporting the current WHO treatment guidelines [39,40].

Of the meningococcal meningitis epidemics in Africa over the past 70 years, 80–85% have been serogroup A. Because of this, these epidemics were often able to be controlled with polysaccharide vaccines. That type of vaccine, however, does not lead to long-lasting immunity, nor does it decrease carriage or transmission [31]. A new conjugate vaccine was developed in 2009 which has been shown to be associated with an impressive decrease in the incidence of meningococcal meningitis as well as *N. meningitides* nasal carriage following a mass immunization campaign in Burkino Faso, Mali, Niger, Chad and areas of Cameroon and Nigeria [41]. In fact, in Chad and Niger, where mass vaccination campaigns have taken place, serogroup A incidence has massively decreased [42,43]. While mass vaccination campaigns are currently underway in the meningitis belt, a recently published model shows that a more sustainable approach to obtain the lowest infection rates would also include follow-up vaccination campaigns of one to five year olds every five years [44].

### Other bacterial pathogens

While there have been reports of other bacterial causes of meningitis in African patients, such as *Haemophilus influenzae*, *Salmonella spp*., *Enterobacter cloacae*, *Escherichia coli*, *Pseudomonas aeruginosa*, *Staphylococcus aureus* and *Klebsiella pneumonia*, they only account for 1–6% of the total reported cases [5,11].

### Fungal meningitis

#### Cryptococcus neoformans

The HIV epidemic has caused a marked increase in cases of cryptococcal meningitis such that it has become the most common cause of meningitis in some areas of sub-Saharan Africa and was the leading cause of meningitis in our review among African patients living with HIV [5,9,45]. Sub-Saharan Africa has the highest incidence of cryptococcal meningitis in the world with an estimated 720,000 new cases reported in 2006 and an estimated 504,000 deaths. When comparing the estimate of deaths in sub-Saharan Africa with other diseases excluding HIV infection, deaths associated with cryptococcal meningitis are higher than those from tuberculosis (350,000), with the former causing 20–25% of AIDS-related mortality in Africa [46]. The cryptococcal meningitis case fatality rate is 35–65% in African patients living with HIV compared to 14–26% amongst patients living with HIV in high-income countries [47–51].

Part of the explanation for the high mortality rate is the limited diagnostic resources available in many health centres across sub-Saharan Africa. There are no clinical criteria that reliably predict the diagnosis of cryptococcal meningitis, and labs often lack the ability to perform fungal cultures or CrAg testing [9]. In 2011, with the advent of new, low-cost, point-of-care lateral flow assays (LFAs), which have very high sensitivity and specificity, WHO recommended a change to the diagnostic work-up of meningitis to include prompt lumbar puncture (LP) with measurement of rapid CSF CrAg or rapid serum CrAg [52–54]. In areas of high HIV prevalence, including CrAg screening in the initial work-up for meningitis has been shown to be highly cost-effective and should be considered [55].

Another cause for the high mortality rate seen in cryptococcal meningitis is limited access to recommended treatment in much of sub-Saharan Africa. A recent randomized controlled trial in Vietnam demonstrated that two-week induction treatment with amphotericin B and flucytosine was associated with improved survival at all endpoints compared to amphotericin alone [56]. However, those antifungal agents are not available in many African medical centres. Amphotericin-based regimens also require rigorous laboratory monitoring, including a minimum of biweekly potassium and creatinine measurements, pre-hydration with 1 L normal saline prior to amphotericin dosing and management of electrolyte abnormalities to maintain potassium levels >3.3. If amphotericin B is not available, high-dose fluconazole plus flucytosine, or high-dose fluconazole alone, is recommended [52]. It is also critical to measure opening pressure when performing LPs. If elevated (>20 cm H_2_O), in order to prevent complications caused by increased intracranial pressure, daily therapeutic LPs should be performed until the opening pressure measurement is normal on more than two consecutive days. Early Initiation of ART early after cryptococcal meningitis diagnosis (<2 weeks) was recently shown to increase overall mortality because of immune reconstitution syndrome; therefore, it is recommended to wait either 2–4 weeks if amphotericin induction is used or 4–6 weeks if fluconazole induction is used prior to ART initiation [52,57,58].

Given the difficulties in managing cryptococcal meningitis along with the high mortality rates, increasing awareness is being given to prevention of this life-threatening disease. Initial attempts to provide primary prophylaxis with fluconazole or itraconazole did not lead to improved mortality outcomes [59]. With the advent of the CrAg LFA of serum, which is a new, low-cost, highly sensitive, point-of-care screening test, a screen-and-treat strategy is now being recommended in HIV-positive patients with CD4<100 cells/µL. This approach has been shown to decrease the incidence in the development of cryptococcal meningitis in asymptomatic patients who receive pre-emptive treatment with fluconazole (800 mg orally ×2 weeks, followed by 400 mg orally ×8 weeks, followed by 200 mg orally daily until CD4>200) after screening positive with the CrAg LFA. Furthermore, this has been found to be a cost-effective approach and is increasingly being implemented across sub-Saharan Africa into routine HIV care for those with CD4<100 cells/µL at initiation of care [52,60–62].

### Other fungal pathogens

Other fungal pathogens rarely involve the meninges. *Histoplasma capsulatum* can occasionally cause meningitis, and it is endemic to Africa, most frequently seen in South Africa [63]. *Candida* species may also rarely cause meningitis. Mucosal candidiasis is a common feature among patients living with HIV; however, a disseminated *Candida* infection leading to meningitis is an extremely rare and late complication of AIDS.

### Mycobacterial meningitis

#### Mycobacterium tuberculosis

Tuberculosis is a common cause of death worldwide, but it is particularly severe amongst patients living with HIV, killing 250,000 African HIV patients in 2012 [64]. HIV-positive patients are more likely to develop extrapulmonary TB (including TB meningitis) compared to HIV-negative patients [65]. *Mycobacterium tuberculosis* is the second leading cause of meningitis in African patients living with HIV, according to our review.

Common symptoms of tuberculous meningitis are fever, headache, irritability, neck stiffness, lethargy, nausea and vomiting, focal neurological signs, altered consciousness, SIADH, stroke, brain abscess and seizures [66]. One study showed increased prevalence of impaired level of consciousness in patients living with HIV [67]; however, in a recent review, the neurologic presentation amongst HIV-positive and HIV-negative patients did not have wide variation. In that review, HIV-positive patients did, however, present with increased weight loss compared to HIV-negative [68]. Symptoms may be very similar to those of cryptococcal meningitis, which makes distinguishing between the two based on clinical presentation alone very difficult and highlights the urgent need to use available rapid diagnostics [9,68]. Diagnosis of tuberculous meningitis is challenging as symptoms are nonspecific and laboratory techniques lack sensitivity. Recently a diagnostic scoring system was proposed for use in research settings. While this score lacks clinical validation, it may assist clinicians in developing a framework for use in the diagnosis of tuberculosis and is summarized in Table 2, [66]. The classic CSF analysis seen in tuberculous meningitis includes a high white blood cell count with lymphocyte predominance, high protein concentration and low glucose concentration. Those findings are not universally seen in all tuberculous meningitis cases, and CSF results may occasionally even be normal [66,69]. In addition, tests for *Mycobacterium tuberculosis* in the CSF are notoriously insensitive as well as slow and expensive. AFB smear of CSF is the traditional point-of-care test, but it has a sensitivity of only about 20% unless higher volume taps (>7 ml) and increased microscopy time (30 minutes) are employed [70]. Liquid culture techniques improve sensitivity to almost 60%, but take 1–4 weeks to finalize [71]. Tests using the biomarker adenosine deaminase (ADA) have not been shown to be sufficiently sensitive or specific for diagnosis [72]. Because of those limitations, there has been increasing interest in new point-of-care tests. The use of nucleic acid amplification tests (NAATs), specifically GeneXpert MTB/RIF (cepheid, Sunnyvale, Ca, USA), is an attractive diagnostic option in resource-limited settings because of its simplicity of use, low rates of cross-contamination, short result time and ability to rapidly detect rifampicin resistance. The main limitation is its high cost. It has been evaluated on CSF samples with varying results (sensitivity, 27–86%; specificity, 99–100%) [73,74]. The lipoarabinomannan (LAM) antigen detection enzyme-linked immunosorbent assay (ELISA) was recently evaluated for use in CSF for diagnosis of tuberculous meningitis. It showed the highest utility when used in HIV-positive patients with CD4 < 100 µL/mm^3^ and when combined with a clinical prediction tool (sensitivity, 63%; 95% CI, 47–68; and specificity, 93%; 95% CI, 82–98). The low cost and point-of-care platform make that test appealing for resource-limited settings [75]. Interferon-gamma release assays are highly sensitive and specific for the diagnosis of latent tuberculosis, but they cannot reliably be used to rule out active disease [76]. Diagnostic imaging such as computed tomography (CT) or magnetic resonance imaging (MRI) can also be used in the diagnosis of tuberculous meningitis, but it is not widely available in sub-Saharan Africa. Radiographic findings include hydrocephalus and/or basal meningeal enhancement with infarcts and tuberculomas, with MRI having higher sensitivity than a CT scan. Those finding are not highly specific and can be seen in other aetiologies as well [66].

All patients living with HIV and tuberculous meningitis require treatment with ART; however, immediate initiation is associated with increased adverse events. It is therefore generally recommended to initiate ART 2–8 weeks after initiation of TB treatment in tuberculous meningitis [77–79]. This is in contrast to treatment for pulmonary tuberculosis, in which recent studies have shown that early initiation of treatment is generally preferred [80]. Treatment of tuberculous meningitis generally consists of rifampicin and isoniazid for 9–12 months plus pyrazinamide with ethambutol, streptomycin or ethionamide for the first two months [68]. The drugs with the highest CSF penetration include isoniazid, pyrazinamide and ethionamide [81]. Fluoroquinolones such as levofloxacin and moxifloxacin also have excellent CSF penetration, and a large clinical trial is underway to evaluate current standard tuberculous meningitis treatment regimens with standard treatment intensified with high-dose rifampicin and additional levofloxacin [82]. Drug-drug interactions must be considered when selecting an ART regimen. Rifampicins significantly reduce concentrations of protease inhibitors, and their concomitant use is relatively contraindicated. Regimens with non-nucleoside reverse transcriptase inhibitors are preferred, and efavirenz (+/− dose modification) is considered the drug of choice because of lower risk of drug-drug interactions [83,84]. The benefit of adjunctive corticosteroid treatment for tuberculous meningitis in individuals living with HIV is generally recommended based on benefit seen in the general population. In a randomized controlled study from Vietnam including 98 HIV-positive and HIV-negative adults, adjunctive treatment with dexamethasone improved the overall probability of survival at nine months and two years but not at five years. Among an HIV-positive subgroup, there was a non-significant reduction in death at nine months. In a Cochrane review, corticosteroid use showed inconclusive benefit in patients living with HIV and tuberculous meningitis [85–87].

Prevention efforts for tuberculosis meningitis in adults are limited. In a meta-analysis, the Bacillus Calmette-Guérin (BCG) vaccine has been found to be highly efficacious in preventing meningitis in children. Age at vaccination was not a significant TB predictor of vaccine efficacy; however, more research is needed to assess its safety as there have been case reports of fatal BCG infections among individuals living with HIV [88–93]. Isoniazid prophylaxis is currently recommended by WHO for all HIV-positive patients with unknown or positive tuberculin skin test (TST) who reside in resource-limited, high-burden countries where active tuberculosis infection has been excluded [94] (Table 3)

**Table 2:** Diagnostic criteria for the classification of definite, probable, possible and not tuberculous meningitis

*Clinical entry criteria*: ≥1 of the following: headache, irritability, vomiting, fever, neck stiffness, convulsions, focal neurological deficits, altered consciousness or lethargy	
*Definite*: (A) Clinical entry criteria+≥1 of the following: AFB seen in the CSF; *Mycobacterium tuberculosis* cultured from the CSF; or a CSF positive commercial nucleic acid amplification test (NAAT) (B) AFB seen in the context of histologic changes consistent with TB in the brain or spinal cord with suggestive symptoms or signs and CSF changes, or visible meningitis (on autopsy)	
*Probable*: Clinical entry criteria+total diagnostic score of ≥10 points (when cerebral imaging is not available) or ≥12 points (when cerebral imaging is available) plus exclusion of alternative diagnoses. At least 2 points should come from either CSF or cerebral-imaging criteria.	
*Possible*: Clinical entry criteria+total diagnostic score of 6–9 points (when cerebral imaging is not available) or 6–11 points (when cerebral imaging is available) plus exclusion of alternative diagnoses. Possible tuberculosis cannot be diagnosed or excluded without doing a lumbar puncture or cerebral imaging.	
*Not tuberculous meningitis*: Alternative diagnosis established, without a definitive diagnosis of tuberculous meningitis or other convincing signs of dual disease.	
Clinical criteria	
Symptom duration >5 days	4
≥ 1 symptom: weight loss, night sweats, persistent cough for more than 2 weeks	2
History of close contact with person with pulmonary tuberculosis within the past year	2
Focal neurological deficit (cranial nerve palsies excluded)	1
Cranial nerve palsy	1
Altered consciousness	1
CSF criteria	
Clear appearance	1
Cells: 10–500/µl	1
Lymphocytic predominance (>50%)	1
Protein concentration >1 g/L	1
CSF-to-plasma glucose ratio of <50% or absolute CSF glucose concentration <2.2 mmol/L	1
Cerebral imaging criteria	
Hydrocephalus	1
Basal meningeal enhancement	2
Tuberculoma	2
Infarct	1
Pre-contrast basal hyperdensity	2
Evidence of tuberculosis elsewhere	
Chest radiograph suggestive of active tuberculosis	2: signs of TB; 4: miliary TB
CT, MRI or ultrasound evidence for tuberculosis outside the CNS	2
AFB identified or *Mycobacterium tuberculosis* cultured from another source	4
Positive commercial *M. tuberculosis* NAAT from extra-neural specimen	4

Source: Adapted from Marais et al. [66].

**Table 3:** Summary of meningitis treatment recommendations from WHO and various studies

Aetiology	Treatment	Dose	Duration
Pneumococcal meningitis	Ceftriaxone ± Dexamethasone	2 g IV q12 0.4 mg/kg q12 hr	7–10 days 4 days
Meningococcal meningitis	Ceftriaxone Chloramphenicol	2 g/day IM or IV 4 g/day IM (max 4 g) 100 mg/kg IM (max 3 g)	5 days 1 dose in epidemic setting 1 dose in epidemic setting
Cryptococcal meningitis	Induction: Amphotericin + Flucytosine If amphotericin not available: Fluconazole ± Flucytosine	0.7–1 mg/kg/day 100 mg/kg/day 1200 mg po qd 100 mg/kg/day	2 weeks if pre-hydration+electrolyte replacement+toxicity monitoring available; otherwise, 5–7 days 2 weeks
	Consolidation: Fluconazole	800 mg po qday	8 weeks
	Maintenance: Fluconazole	200 mg po qday	Continue until: -stable AND -adherent to ART and fluconazole for ≥1 year AND -CD4 cell count ≥200 cells/mm^3^ (measured twice, 6 months apart).
Tuberculous meningitis	Rifampicin + Isoniazid + Pyrazinamide + either Ethambutol, Ethionamide or Streptomycin + Dexamethasone	8–12 mg/kg (max 600 mg) po qday 4–6 mg/k (max 300 mg) po qday 20–30 mg/kg po qday 15–20 mg/kg po qday 15–20 mg/kg po qday (max 1 g/d) 12–18 mg/kg po qday 0.3–0.4 mg/kg/day ×2 weeks, then 0.2 mg/kg/day ×2 weeks, then 4 mg per day and taper 1 mg off qwk	9–12 months 9–12 months For the first 2 months For the first 2 months Total duration: 8 weeks

Sources: From Refs. [18,20,52,84,95].

. Clinical benefits of isoniazid prophylaxis were seen in those HIV-positive patients with positive TST, with a 74% (*p =* 0.02) reduction in TB incidence and a 68% (95% CI, 0.11–0.90) mortality reduction. That reduction was seen while taking isoniazid; however, these results were not durable after isoniazid was discontinued [96]. Because of the concerns of toxicity with unclear benefit in those patients with negative TST, as well as unnecessary cost, drug-drug interactions and difficulty in diagnosing active TB in HIV-positive patients, routine use of isoniazid prophylaxis has not been universally practiced despite being widely recommended.

### Other causes of meningitis

Viral meningitis was rarely seen in studies, although prevalence may be higher than reported as diagnostics are often limited. Acute retroviral syndrome is also a known cause of meningitis and generally recovers as the acute viraemia resolves [97,98].

Aseptic meningitis has been described that results from trimethoprim-sulphamethoxazole and other drugs. It presents as acute meningitis with lymphocytic pleocytosis, and prompt resolution is seen when the drug is stopped [99].

## Conclusions

Meningitis remains a major cause of morbidity and mortality among patients living with HIV in sub-Saharan Africa, and in many cases it is potentially preventable. This study shows that the aetiology of meningitis among people living with HIV in sub-Saharan Africa is largely composed of cryptococcal meningitis, followed by tuberculous meningitis and bacterial meningitis. These findings are limited by the small number of studies found and the limited reporting systems in place for this population. More work is needed to further develop the reporting among this population on a larger scale.

Diagnosis is often limited, and wider availability of accurate and low-cost laboratory diagnostics is desperately needed for prompt diagnosis and initiation of appropriate treatment. Strong consideration should be given to make pneumococcal conjugate vaccine and cryptococcal screening a part of routine HIV care. Continued support of implementation of wide-scale antiretroviral treatment is also critical. Wider acceptance and adoption of available preventative modalities can decrease the incidence of potentially fatal central nervous system infections in African patients living with HIV.

## References

[1] Walker AS, Prendergast AJ, Mugyenyi P, Munderi P, Hakim J, Kekitiinwa A (2012). Mortality in the year following antiretroviral therapy initiation in HIV-infected adults and children in Uganda and Zimbabwe. Clin Infect Dis.

[2] Lawn SD, Harries AD, Wood R (2010). Strategies to reduce early morbidity and mortality in adults receiving antiretroviral therapy in resource-limited settings. Curr Opin HIV AIDS.

[3] Bergemann A, Karstaedt AS (1996). The spectrum of meningitis in a population with high prevalence of HIV disease. QJM.

[4] Schutte CM, Van der Meyden CH, Magazi DS (2000). The impact of HIV on meningitis as seen at a South African Academic Hospital (1994 to 1998). Infection.

[5] Bekondi C, Bernede C, Passone N, Minssart P, Kamalo C, Mbolidi D (2006). Primary and opportunistic pathogens associated with meningitis in adults in Bangui, Central African Republic, in relation to human immunodeficiency virus serostatus. Int J Infect Dis.

[6] Hakim JG, Gangaidzo IT, Heyderman RS, Mielke J, Mushangi E, Taziwa A (2000). Impact of HIV infection on meningitis in Harare, Zimbabwe: a prospective study of 406 predominantly adult patients. AIDS.

[7] Silber E, Sonnenberg P, Ho KC, Koornhof HJ, Eintracht S, Morris L (1999). Meningitis in a community with a high prevalence of tuberculosis and HIV infection. J Neurol Sci.

[8] Jarvis JN, Meintjes G, Williams A, Brown Y, Crede T, Harrison TS (2010). Adult meningitis in a setting of high HIV and TB prevalence: findings from 4961 suspected cases. BMC Infect Dis.

[9] Cohen DB, Zijlstra EE, Mukaka M, Reiss M, Kamphambale S, Scholing M (2010). Diagnosis of cryptococcal and tuberculous meningitis in a resource-limited African setting. Trop Med Int Health.

[10] World Health Organization (2005). Weekly epidemiological record: enhanced surveillance of epidemic meningococcal meningitis in Africa: a three-year experience.

[11] Nkoumou MO, Betha G, Kombila M, Clevenbergh P (2003). Bacterial and mycobacterial meningitis in HIV-positive compared with HIV-negative patients in an internal medicine ward in Libreville, Gabon. J Acquir Immune Defic Syndr.

[12] Centers for Disease Control (2012). Use of 13-valent pneumococcal conjugate vaccine and 23-valent pneumococcal polysaccharide vaccine for adults with immunocompromising conditions: recommendations of the Advisory Committee on Immunization Practices (ACIP). MMWR Morb Mortal Wkly Rep.

[13] Almirante B, Saballs M, Ribera E, Pigrau C, Gavalda J, Gasser I (1998). Favorable prognosis of purulent meningitis in patients infected with human immunodeficiency virus. Clin Infect Dis.

[14] Wall EC, Cartwright K, Scarborough M, Ajdukiewicz KM, Goodson P, Mwambene J (2013). High mortality amongst adolescents and adults with bacterial meningitis in sub-Saharan Africa: an analysis of 715 cases from Malawi. PLoS One.

[15] Manga NM, Ndour CT, Diop SA, Ka-Sall R, Dia NM, Seydi M (2008). [Adult purulent meningitis caused by Streptococcus pneumoniae in Dakar, Senegal]. Med Trop (Mars).

[16] van de Beek D, de Gans J, Spanjaard L, Weisfelt M, Reitsma JB, Vermeulen M (2004). Clinical features and prognostic factors in adults with bacterial meningitis. N Engl J Med.

[17] Weisfelt M, van de Beek D, Spanjaard L, Reitsma JB, de Gans J (2006). Clinical features, complications, and outcome in adults with pneumococcal meningitis: a prospective case series. Lancet Neurol.

[18] World Health Organization (2007). Standardized treatment of bacterial meningitis in Africa in epidemic and non epidemic situations, 2007.

[19] Ginsburg AS, Tinkham L, Riley K, Kay NA, Klugman KP, Gill CJ (2013). Antibiotic non-susceptibility among Streptococcus pneumoniae and Haemophilus influenzae isolates identified in African cohorts: a meta-analysis of three decades of published studies. Int J Antimicrob Agents.

[20] Nguyen TH, Tran TH, Thwaites G, Ly VC, Dinh XS, Ho Dang TN (2007). Dexamethasone in Vietnamese adolescents and adults with bacterial meningitis. N Engl J Med.

[21] Borchorst S, Moller K (2012). The role of dexamethasone in the treatment of bacterial meningitis – a systematic review. Acta Anaesthesiol Scand.

[22] Brouwer MC, McIntyre P, Prasad K, van de Beek D (2013). Corticosteroids for acute bacterial meningitis. Cochrane Database Syst Rev.

[23] Scarborough M, Gordon SB, Whitty CJ, French N, Njalale Y, Chitani A (2007). Corticosteroids for bacterial meningitis in adults in sub-Saharan Africa. N Engl J Med.

[24] Ajdukiewicz KM, Cartwright KE, Scarborough M, Mwambene JB, Goodson P, Molyneux ME (2011). Glycerol adjuvant therapy in adults with bacterial meningitis in a high HIV seroprevalence setting in Malawi: a double-blind, randomised controlled trial. Lancet Infect Dis.

[25] French N, Nakiyingi J, Carpenter LM, Lugada E, Watera C, Moi K (2000). 23-valent pneumococcal polysaccharide vaccine in HIV-1-infected Ugandan adults: double-blind, randomised and placebo controlled trial. Lancet.

[26] Gebo KA, Moore RD, Keruly JC, Chaisson RE (1996). Risk factors for pneumococcal disease in human immunodeficiency virus-infected patients. J Infect Dis.

[27] Rodriguez-Barradas MC, Goulet J, Brown S, Goetz MB, Rimland D, Simberkoff MS (2008). Impact of pneumococcal vaccination on the incidence of pneumonia by HIV infection status among patients enrolled in the Veterans Aging Cohort 5-Site Study. Clin Infect Dis.

[28] Nunes MC, Madhi SA (2012). Safety, immunogenicity and efficacy of pneumococcal conjugate vaccine in HIV-infected individuals. Hum Vaccin Immunother.

[29] French N, Gordon SB, Mwalukomo T, White SA, Mwafulirwa G, Longwe H (2010). A trial of a 7-valent pneumococcal conjugate vaccine in HIV-infected adults. N Engl J Med.

[30] World Health Organization (2013). WHO advisory committee on immunization and vaccine related implementation research (IVIR, formerly QUIVER): executive summary report of 7th meeting.

[31] Greenwood B (2006). Editorial: 100 years of epidemic meningitis in West Africa – has anything changed?. Trop Med Int Health.

[32] Greenwood BM, Bradley AK, Wall RA (1985). Meningococcal disease and season in sub-Saharan Africa. Lancet.

[33] Brindle R, Simani P, Newnham R, Waiyaki P, Gilks C (1991). No association between meningococcal disease and human immunodeficiency virus in adults in Nairobi, Kenya. Trans R Soc Trop Med Hyg.

[34] Gordon SB, Walsh AL, Chaponda M, Gordon MA, Soko D, Mbwvinji M (2000). Bacterial meningitis in Malawian adults: pneumococcal disease is common, severe, and seasonal. Clin Infect Dis.

[35] Batchelor BI, Kimari JN, Brindle RJ (1996). Microbiology of HIV associated bacteraemia and diarrhoea in adults from Nairobi, Kenya. Epidemiol Infect.

[36] Pallangyo K, Hakanson A, Lema L, Arris E, Mteza I, Palsson K (1992). High HIV seroprevalence and increased HIV-associated mortality among hospitalized patients with deep bacterial infections in Dar es Salaam, Tanzania. AIDS.

[37] Cohen C, Singh E, Wu HM, Martin S, de Gouveia L, Klugman KP (2010). Increased incidence of meningococcal disease in HIV-infected individuals associated with higher case-fatality ratios in South Africa. AIDS.

[38] Veeken H, Ritmeijer K, Hausman B (1998). Priority during a meningitis epidemic: vaccination or treatment?. Bull World Health Organ.

[39] Hedberg ST, Fredlund H, Nicolas P, Caugant DA, Olcen P, Unemo M (2009). Antibiotic susceptibility and characteristics of Neisseria meningitidis isolates from the African meningitis belt, 2000 to 2006: phenotypic and genotypic perspectives. Antimicrob Agents Chemother.

[40] World Health Organization (2012). Meningococcal meningitis fact sheet.

[41] Kristiansen PA, Diomande F, Ba AK, Sanou I, Ouedraogo AS, Ouedraogo R (2013). Impact of the serogroup A meningococcal conjugate vaccine, MenAfriVac, on carriage and herd immunity. Clin Infect Dis.

[42] Daugla DM, Gami JP, Gamougam K, Naibei N, Mbainadji L, Narbe M (2014). Effect of a serogroup A meningococcal conjugate vaccine (PsA-TT) on serogroup A meningococcal meningitis and carriage in Chad: a community study [corrected]. Lancet.

[43] Collard JM, Issaka B, Zaneidou M, Hugonnet S, Nicolas P, Taha MK (2013). Epidemiological changes in meningococcal meningitis in Niger from 2008 to 2011 and the impact of vaccination. BMC Infect Dis.

[44] Tartof S, Cohn A, Tarbangdo F, Djingarey MH, Messonnier N, Clark TA (2013). Identifying optimal vaccination strategies for serogroup A Neisseria meningitidis conjugate vaccine in the African meningitis belt. PLoS One.

[45] Bisson GP, Lukes J, Thakur R, Mtoni I, MacGregor RR (2008). Cryptococcus and lymphocytic meningitis in Botswana. S Afr Med J.

[46] Park BJ, Wannemuehler KA, Marston BJ, Govender N, Pappas PG, Chiller TM (2009). Estimation of the current global burden of cryptococcal meningitis among persons living with HIV/AIDS. AIDS.

[47] Lessells RJ, Mutevedzi PC, Heller T, Newell ML (2011). Poor long-term outcomes for cryptococcal meningitis in rural South Africa. S Afr Med J.

[48] Kambugu A, Meya DB, Rhein J, O'Brien M, Janoff EN, Ronald AR (2008). Outcomes of cryptococcal meningitis in Uganda before and after the availability of highly active antiretroviral therapy. Clin Infect Dis.

[49] Lortholary O, Poizat G, Zeller V, Neuville S, Boibieux A, Alvarez M (2006). Long-term outcome of AIDS-associated cryptococcosis in the era of combination antiretroviral therapy. AIDS.

[50] Saag MS, Powderly WG, Cloud GA, Robinson P, Grieco MH, Sharkey PK (1992). Comparison of amphotericin B with fluconazole in the treatment of acute AIDS-associated cryptococcal meningitis. The NIAID Mycoses Study Group and the AIDS Clinical Trials Group. N Engl J Med.

[51] Robinson PA, Bauer M, Leal MA, Evans SG, Holtom PD, Diamond DA (1999). Early mycological treatment failure in AIDS-associated cryptococcal meningitis. Clin Infect Dis.

[52] World Health Organization (2011). Rapid advice: diagnosis, prevention and management of cryptococcal disease in HIV-infected adults, adolescents and children.

[53] Kabanda T, Siedner MJ, Klausner JD, Muzoora C, Boulware DR (2014). Point-of-care diagnosis and prognostication of cryptococcal meningitis with the cryptococcal antigen lateral flow assay on cerebrospinal fluid. Clin Infect Dis.

[54] Rugemalila J, Maro VP, Kapanda G, Ndaro AJ, Jarvis JN (2013). Cryptococcal antigen prevalence in HIV-infected Tanzanians: a cross-sectional study and evaluation of a point-of-care lateral flow assay. Trop Med Int Health.

[55] Durski KN, Kuntz KM, Yasukawa K, Virnig BA, Meya DB, Boulware DR (2013). Cost-effective diagnostic checklists for meningitis in resource-limited settings. J Acquir Immune Defic Syndr.

[56] Day JN, Chau TT, Lalloo DG (2013). Combination antifungal therapy for cryptococcal meningitis. N Engl J Med.

[57] Boulware D, Meya D, Muzoora C, Rolfes M, Huppler Hullsiek K, Musubire A (2013). ART initiation within the first 1 weeks of cryptococcal meningitis is associated with higher mortality: a multisite randomized trial.

[58] Makadzange AT, Ndhlovu CE, Takarinda K, Reid M, Kurangwa M, Gona P (2010). Early versus delayed initiation of antiretroviral therapy for concurrent HIV infection and cryptococcal meningitis in sub-Saharan Africa. Clin Infect Dis.

[59] Chang LW, Phipps WT, Kennedy GE, Rutherford GW (2005). Antifungal interventions for the primary prevention of cryptococcal disease in adults with HIV. Cochrane Database Syst Rev.

[60] Micol R, Tajahmady A, Lortholary O, Balkan S, Quillet C, Dousset JP (2010). Cost-effectiveness of primary prophylaxis of AIDS associated cryptococcosis in Cambodia. PLoS One.

[61] Jarvis JN, Govender N, Chiller T, Park BJ, Longley N, Meintjes G (2012). Cryptococcal antigen screening and preemptive therapy in patients initiating antiretroviral therapy in resource-limited settings: a proposed algorithm for clinical implementation. J Int Assoc Physicians AIDS Care (Chic).

[62] Rajasingham R, Meya DB, Boulware DR (2012). Integrating cryptococcal antigen screening and pre-emptive treatment into routine HIV care. J Acquir Immune Defic Syndr.

[63] Gugnani HC (2000). Histoplasmosis in Africa: a review. Indian J Chest Dis Allied Sci.

[64] World Health Organization (2012). Global tuberculosis control: regional profiles.

[65] Marais S, Pepper DJ, Schutz C, Wilkinson RJ, Meintjes G (2011). Presentation and outcome of tuberculous meningitis in a high HIV prevalence setting. PLoS One.

[66] Marais S, Thwaites G, Schoeman JF, Torok ME, Misra UK, Prasad K (2010). Tuberculous meningitis: a uniform case definition for use in clinical research. Lancet Infect Dis.

[67] Katrak SM, Shembalkar PK, Bijwe SR, Bhandarkar LD (2000). The clinical, radiological and pathological profile of tuberculous meningitis in patients with and without human immunodeficiency virus infection. J Neurol Sci.

[68] Marais S, Pepper DJ, Marais BJ, Torok ME (2010). HIV-associated tuberculous meningitis – diagnostic and therapeutic challenges. Tuberculosis (Edinb).

[69] Karstaedt AS, Valtchanova S, Barriere R, Crewe-Brown HH (1998). Tuberculous meningitis in South African urban adults. QJM.

[70] Thwaites GE, Chau TT, Farrar JJ (2004). Improving the bacteriological diagnosis of tuberculous meningitis. J Clin Microbiol.

[71] Caws M, Dang TM, Torok E, Campbell J, Do DA, Tran TH (2007). Evaluation of the MODS culture technique for the diagnosis of tuberculous meningitis. PLoS One.

[72] Tuon FF, Higashino HR, Lopes MI, Litvoc MN, Atomiya AN, Antonangelo L (2010). Adenosine deaminase and tuberculous meningitis – a systematic review with meta-analysis. Scand J Infect Dis.

[73] Vadwai V, Boehme C, Nabeta P, Shetty A, Alland D, Rodrigues C (2011). Xpert MTB/RIF: a new pillar in diagnosis of extrapulmonary tuberculosis?. J Clin Microbiol.

[74] Tortoli E, Russo C, Piersimoni C, Mazzola E, Dal Monte P, Pascarella M (2012). Clinical validation of Xpert MTB/RIF for the diagnosis of extrapulmonary tuberculosis. Eur Respir J.

[75] Patel VB, Singh R, Connolly C, Kasprowicz V, Zumla A, Ndungu T (2010). Comparison of a clinical prediction rule and a LAM antigen-detection assay for the rapid diagnosis of TBM in a high HIV prevalence setting. PLoS One.

[76] Rangaka MX, Wilkinson KA, Glynn JR, Ling D, Menzies D, Mwansa-Kambafwile J (2012). Predictive value of interferon-gamma release assays for incident active tuberculosis: a systematic review and meta-analysis. Lancet Infect Dis.

[77] Torok ME, Yen NT, Chau TT, Mai NT, Phu NH, Mai PP (2011). Timing of initiation of antiretroviral therapy in human immunodeficiency virus (HIV) – associated tuberculous meningitis. Clin Infect Dis.

[78] Bicanic TA, Jarvis JN, Loyse A, Harrison TS (2013). Starting ART following cryptococcal meningitis: the optimal time has yet to be defined. South Afr J HIV Med.

[79] Meintjes G, Maartens G, Boulle A, Conradie F, Goemaere E, Hefer E (2012). Guidelines for antiretroviral therapy in adults by the Southern African HIV Clinicians Society. SAJHIVMED.

[80] Blanc FX, Sok T, Laureillard D, Borand L, Rekacewicz C, Nerrienet E (2011). Earlier versus later start of antiretroviral therapy in HIV-infected adults with tuberculosis. N Engl J Med.

[81] Thwaites GE, van Toorn R, Schoeman J (2013). Tuberculous meningitis: more questions, still too few answers. Lancet Neurol.

[82] Heemskerk D, Day J, Chau TT, Dung NH, Yen NT, Bang ND (2011). Intensified treatment with high dose rifampicin and levofloxacin compared to standard treatment for adult patients with tuberculous meningitis (TBM-IT): protocol for a randomized controlled trial. Trials.

[83] Zumla A, Raviglione M, Hafner R, von Reyn CF (2013). Tuberculosis. N Engl J Med.

[84] World Health Organization (2010). Treatment of tuberculosis: guidelines.

[85] Torok ME, Nguyen DB, Tran TH, Nguyen TB, Thwaites GE, Hoang TQ (2011). Dexamethasone and long-term outcome of tuberculous meningitis in Vietnamese adults and adolescents. PLoS One.

[86] Prasad K, Singh MB (2008). Corticosteroids for managing tuberculous meningitis. Cochrane Database Syst Rev.

[87] Volkow PF, Cornejo P, Zinser JW, Ormsby CE, Reyes-Teran G (2008). Life-threatening exacerbation of Kaposi's sarcoma after prednisone treatment for immune reconstitution inflammatory syndrome. AIDS.

[88] Colditz GA, Brewer TF, Berkey CS, Wilson ME, Burdick E, Fineberg HV (1994). Efficacy of BCG vaccine in the prevention of tuberculosis. Meta-analysis of the published literature. JAMA.

[89] Albrecht H, Stellbrink HJ, Eggers C, Rusch-Gerdes S, Greten H (1995). A case of disseminated *Mycobacterium bovis* infection in an AIDS patient. Eur J Clin Microbiol Infect Dis.

[90] Centers for Disease Control (1985). Disseminated *Mycobacterium bovis* infection from BCG vaccination of a patient with acquired immunodeficiency syndrome. MMWR Morb Mortal Wkly Rep.

[91] Reynes J, Perez C, Lamaury I, Janbon F, Bertrand A (1989). Bacille Calmette-Guerin adenitis 30 years after immunization in a patient with AIDS. J Infect Dis.

[92] Weltman AC, Rose DN (1993). The safety of Bacille Calmette-Guerin vaccination in HIV infection and AIDS. AIDS.

[93] Boudes P, Sobel A, Deforges L, Leblic E (1989). Disseminated Mycobacterium bovis infection from BCG vaccination and HIV infection. JAMA.

[94] World Health Organization (2011). Guidelines for intensified tuberculosis case-finding and isoniazid preventive therapy for people living with HIV in resource-constrained settings.

[95] Thwaites GE, Nguyen DB, Nguyen HD, Hoang TQ, Do TT, Nguyen TC (2004). Dexamethasone for the treatment of tuberculous meningitis in adolescents and adults. N Engl J Med.

[96] Samandari T, Agizew TB, Nyirenda S, Tedla Z, Sibanda T, Shang N (2011). 6-month versus 36-month isoniazid preventive treatment for tuberculosis in adults with HIV infection in Botswana: a randomised, double-blind, placebo-controlled trial. Lancet.

[97] Huang ST, Lee HC, Liu KH, Lee NY, Ko WC (2005). Acute human immunodeficiency virus infection. J Microbiol Immunol Infect.

[98] Li CM, Lee YY, Ho YR (2002). Acute meningoencephalitis as initial presentation of human immunodeficiency virus infection: report of two cases. J Microbiol Immunol Infect.

[99] Jurado R, Carpenter SL, Rimland D (1996). Case reports: trimethoprim-sulfamethoxazole-induced meningitis in patients with HIV infection. Am J Med Sci.

